# Urban–rural differences in health literacy in the metropolitan area of Berlin, Germany, and its surroundings

**DOI:** 10.1093/eurpub/ckad070

**Published:** 2023-05-12

**Authors:** Christine Haeger, Sonia Lech, Melanie Messer, Paul Gellert

**Affiliations:** Institute of Medical Sociology and Rehabilitation Science, Charité—Universitätsmedizin Berlin, D-10117 Berlin, Germany; Institute of Medical Sociology and Rehabilitation Science, Charité—Universitätsmedizin Berlin, D-10117 Berlin, Germany; Department of Psychiatry and Psychotherapy, Charité—Universitätsmedizin Berlin, D-10117 Berlin, Germany; Department of Nursing Science II, Trier University, D-54296 Trier, Germany; Institute of Medical Sociology and Rehabilitation Science, Charité—Universitätsmedizin Berlin, D-10117 Berlin, Germany

## Abstract

**Background:**

Health literacy is gaining importance as it concerns the ability of individuals to encounter the complex demands of health in modern societies. Little is known about the environmental associations of health literacy in high-income countries. This study aims to (i) analyse urban–rural differences in health literacy and further (ii) investigate the interrelations of associated factors.

**Methods:**

Based on secondary analyses using a population-based survey of individuals aged 35 years and older from Berlin, Germany, and the surrounding rural area. Health literacy, sociodemographic factors (gender, age, educational level, marital status, income), environmental factors (urban/rural) and health behaviour (physical activity) were assessed with questionnaires. *T*-tests, Analyses of Variance and multiple regression with interaction terms have been applied.

**Results:**

In total, 1822 participants (51.2% female and 56.8% living in an urban region) took part in this study. Health literacy was significantly higher in rural regions (mean = 35.73, SD = 7.56) than in urban regions (mean = 34.10, SD = 8.07). Multiple multivariate regressions showed that living in urban regions, being older, having vocational or basic education, having mid or low income, being widowed, having moderate or low levels of physical activity were significantly negatively associated with health literacy. Incorporating interaction terms showed significance that being older is positively associated with urban regions.

**Conclusion:**

We found higher levels of health literacy in rural regions and also demonstrated that multiple associated factors of health literacy work congruously. Thus, the environment, associated factors and their interplay must be considered in future urban–rural health literacy research.

## Introduction

In modern societies, there is a plethora of knowledge and information, that is easily accessible and always on hand, which is especially true for the increasing availability of online health information^1^ and the rapidly evolving platforms based on Large Language Models such as the chatbot ChatGPT (generated pre-trained).^2^ During COVID-19 pandemic, there has also been a phenomenon called infodemic (short for information epidemic)—a surplus of both valid and invalid information^3^—impeding the ability to make well-informed health decisions. Within this process, health literacy plays a major role and Nutbeam calls for it as a midstream determinant of health.^4^

Health literacy is ‘linked to literacy and entails people’s knowledge, motivation and competences to access, understand, appraise, and apply health information in order to make judgments and take decisions in everyday life concerning healthcare, disease prevention, and health promotion to maintain or improve quality of life during the life course’ (p. 3).^4^ It is associated with educational attainment and general literacy and leading experts and policymakers frequently have pointed out that enhancing health literacy within the population is key to a healthy society.^5^ As a foundation and theoretical classification, the integrated model of Sørensen et al.^6^ is well-known and gained importance as it not only includes personal but also social and environmental determinants. The health literacy model further distinguishes between four steps of information processing which are accessing health information, understanding health information, appraising health information and applying health information.

### Health literacy and health outcomes

There is a growing body of research suggesting that high levels of health literacy are positively associated with health outcomes such as well-being,^7^ access to healthcare^8^ and overall health.^9^ More recently, the concept of health literacy has attracted more attention from governments with the ongoing COVID-19 pandemic situation. For example, Schaeffer et al. (2021) found that levels of health literacy improved slightly within the German population during the COVID-19 pandemic, albeit being still at a non-satisfying rate with 60% of the population having low levels of health literacy.^10^ Whilst higher levels of health literacy were associated with positive health outcomes, the opposite is observed when it comes to having low levels of health literacy. A systematic review by Berkman and colleagues found that lower levels of health literacy are associated with increased hospitalizations, poorer health-related knowledge and comprehension, differential use of healthcare services, and overall lower health and increased mortality in old age.^11^ Thus, it is of great importance to examine the factors that are associated with higher levels of health literacy in order to tailor health literacy-enhancing interventions to the needs of specific populations.

### The association between health literacy, sociodemographic factors and health behaviour

The link between health literacy and sociodemographic determinants has been examined in the past. For example, results from both waves of the Health Literacy Survey Germany, a representative quantitative survey from Germany reported that health literacy is associated, among others, with age, gender and education.^10^^,^^12^ Other empirical work has confirmed a positive association between health literacy and female gender,^13^ higher educational levels and higher income.^14^ Further, marital status appears to be associated with health literacy as divorced, widowed or separated individuals were found to have lower levels of health literacy.^9^^,^^15^^,^^16^ Aside from sociodemographic determinants, attention has been drawn to health risk behaviour such as smoking, drinking and low physical activity and their link to health literacy.^17^ For example, health literacy was found to be protective of smoking, regular drinking and lack of physical activity.^9^^,^^18^^,^^19^ Further, a systematic review of health literacy by Buja et al. found a significant positive link between health literacy and levels of physical activity.^20^

### Health literacy and urban–rural differences

Due to the existence of health inequalities in rural areas,^21^ prior studies have examined urban–rural differences with regard to health literacy. A recent systematic review covering 19 studies found urban–rural differences with higher levels of health literacy in urban regions in all but one study. However, once considering other sociodemographic variables just over 50% of the studies authors came to the point that rurality itself is not a significant determinant of health literacy.^22^ There have also been other studies not part of the systematic review that came to comparable results,^23^^,^^24^ both conducted in Iran. However, all of the aforementioned studies were conducted in the USA, Africa and Asia. To our knowledge, no studies analysing urban–rural differences in health literacy were conducted in Europe. Further, no studies examined the interaction effects between regions (e.g. urban/rural) and associated factors such as sociodemographic factors and health behaviour. To fill this research gap, it is of particular interest to investigate the role of region and health literacy in a European, high-income country. Finally, evidence is sparse concerning the interplay of sociodemographic factors, health behaviour, environmental factors (i.e. region) and their relation with health literacy.

### Aim of the present study

The aim of the present study is to gain insights into the relationship between health literacy and associated factors such as sociodemographic factors, environmental factors (urban/rural) and health behaviour as well as the interplay between those. In particular, the present study focuses on the role of region. Drawing on previous evidence, that region (i.e. rural region) alone does not explain urban–rural differences in health literacy, we aim at examining interaction effects between rural regions and sociodemographic factors and health behaviour. We propose that levels of health literacy are lower in rural regions (Hypothesis 1). Subsequently, we explore urban–rural differences across different processes of health literacy. Further, we propose that there are significant interaction effects of rural regions with other sociodemographic factors and health behaviour (i.e. gender × region age × region, educational level × region, income × region, marital status × region and physical activity × region) regarding the health literacy levels (Hypothesis 2).

## Methods

Data were drawn from the cross-sectional Pfizer Monitor II study conducted in October and November 2016 which was part of a series of ad-hoc surveys concerning health literacy and health knowledge (Pfizer Deutschland GmbH in cooperation with Charité—Universitätsmedizin Berlin). The survey was conducted in Berlin—the capital of Germany with metropolitan character—and Brandenburg, which is the surrounding federal state. Brandenburg is known for vast forests, lakes and farming land, and is predominantly rural, although there are also densely populated areas. A population-based sample of 2000 individuals aged 35 years and above from Berlin (*N* = 1000) and its mostly rural, suburban and urban surroundings in Brandenburg (*N* = 1000) participated in this study. Sampling was stratified on urban/rural, gender, age and education. Written informed consent was obtained from each participant. For the recruitment process and more details on the study design see Salm et al.^25^

### Measures

#### Health literacy

Health literacy was measured using the short version (HLS-EU-Q16)^26^ of the EU-HL-Q47.^14^ An index was calculated using the formula Index = mean(per Item) − 1) × 50/3 yielding a maximum score of 50. Questionnaires were included when at least 14 items have been answered as recommended by Röthlin and Pelikan.^26^ There are also four subcategories indicating the four steps of information processing: ‘finding health information, understanding health information, appraising health information and applying health information.’ Furthermore, the three domains ‘health care, disease prevention, and health promotion’ are incorporated into this scale. The reliability analysis within our study for internal consistency showed satisfying effect with Cronbach’s alpha = 0.89.

#### Region

Region was assessed with the first three digits of the ZIP code. Based on responses, a dichotomous variable (urban = 1, rural = 0) was created. All responders from Berlin (i.e. capital of Germany, 3.7 million inhabitants) and Potsdam (i.e. large city in Brandenburg, 180 000 inhabitants) were categorized as urban, and all other responders were categorized as rural with most participants living in villages and smaller cities of up to 10 000 inhabitants. This classification is in line with the Eurostat-Degree of Urbanisation (DEGURBA), a European-wide classification system for Local Administrative Units (LAU) that describes three types of areas: densely (LAU1), intermediate (LAU2) and thinly populated areas (LAU3).^27^ For our study, all LAU1 and LAU2 areas are coded as ‘urban’ and LAU3 areas as ‘rural’.

#### Education

Education was classified by using the International Classification of Education (ISCED)^28^ with ‘basic’ education meaning ISCED levels 1 and 2, ‘vocational’ ISCED being levels 3 and 4 and ‘degree’ being ISCED levels 5 and above.

#### Income

Income status was obtained by asking about the net household income in seven categories (from ‘less than 700 Euro’ up to ‘more than 3600 Euro’ per month). The data were then pooled and divided in thirds to get three categories leading to: low income (less than €2100), mid income (more than 2100 and less than €3100) and high income (more than €3100).

#### Marital status

For marital status, the following categories were obtained: married, in a relationship, divorced, widowed and single. There was no option for others.

#### Physical activity

Physical activity was subjectively assessed with the International Physical Activity Questionnaire (IPAQ) using the four-item short version.^29^ The responses are transformed according to the official scoring protocol and are categorized in low, moderate and high levels of physical activity.^30^

### Data analysis

In the first step, differences between urban and rural groups were analysed using *t*-tests and Analyses of Variance. For the steps of information processing (i.e. subscales of the HLS-EU-Q16) mean levels were analysed and compared across rural/urban regions. Multiple regressions were computed to analyse the relationship between health literacy and its associated factors (i.e. region, gender, age, educational level, income, marital status and physical activity). Two models were specified: The first with health literacy score being the dependent variable and all of the associated factors as independent variable. In the next step, interaction terms were introduced to the model to test the relational association of region in combination with other associated factors and health literacy. The second model therefore includes the interaction terms gender × region, age × region, education × region, marital status × region, income × region and physical activity × region. Since education, income, marital status and physical activity are categorical variables, interaction terms were created with all categories except for one reference category. Age as a metric variable was centred before building the interaction term. All tests of significance were evaluated based on a *P *<* *0.05 level and confidence interval of 95%. All analyses were performed using SPSS v 27 (IBM Corp., Armonk, NY).

## Results

### Sociodemographic characteristics

Sociodemographic characteristics of the study population reported for urban and rural participants are presented in table 1. Data of 1822 participants were included in this analysis as 178 participants were excluded from the final analyses because they did not indicate where they lived. Of the 1822 participants, 51.2% of the study population were female and 56.8% lived in an urban region. The age ranged from 35 years to 91 years (mean = 56.96, SD = 13.71) and more than half of the participants had a vocational level of education (i.e. ISCED level 3 or 4). Most were married or in a relationship and just over half reported low levels of physical activity.

**Table 1:** Sociodemographic characteristics of urban versus rural participants

Variables	Total (*N *=* *1822)	Urban participants (*N *=* *1036)	Rural participants (*N *=* *786)
	*N* (%)	*N* (%)	*N* (%)
Gender			
Male	889 (48.8)	507 (48.9)	382 (48.6)
Female	933 (51.2)	529 (51.0)	404 (51.3)
Age group			
35–44	416 (22.8)	238 (22.9)	178 (22.6)
45–54	456 (25.0)	258 (24.9)	198 (25.1)
55–64	375 (20.6)	229 (22.1)	146 (18.6)
65–74	329 (18.1)	183 (17.6)	146 (18.6)
75+	246 (13.5)	128 (12.3)	118 (15.0)
Educational level			
Basic	306 (16.8)	160 (15.4)	146 (18.5)
Vocational	1132 (62.2)	654 (63.2)	478 (60.8)
Degree	381 (20.9)	220 (21.2)	161 (20.5)
Marital status			
Married	1127 (61.9)	671 (64.8)	456 (58.0)
Relationship	253 (13.9)	128 (12.4)	125 (15.9)
Divorced	149 (8.2)	85 (8.2)	64 (8.1)
Widowed	155 (8.5)	87 (8.4)	68 (8.7)
Single	138 (7.6)	65 (6.3)	73 (9.3)
Income			
Low	751 (41.3)	427 (41.2)	324 (41.2)
Mid	687 (37.7)	394 (38.0)	293 (37.3)
High	384 (21.1)	215 (20.8)	169 (21.5)
Physical activity			
Low	947 (52.0)	482 (46.5)	465 (59.2)
Moderate	474 (26.0)	316 (30.5)	158 (20.1)
High	401 (22.0)	283 (23.0)	163 (20.7)

Sociodemographic characteristics of German participants (*n* = 1822) aged 35 years and older are presented for urban and rural participants with number of participants (*N*) and percentage (%).

### Rural–urban differences in health literacy

Rural–urban differences in health literacy are presented in table 2. Across regions, the mean level of health literacy was 34.79 (SD = 7.93) with the score being significantly higher in rural regions (*P *<* *0.001). Further, we found significant health literacy variations in age, educational level, marital status income and physical activity levels across the regions (all *P *<* *0.001). Within the urban region, there were significant differences across the variables in age, educational level income and physical activity; whereas in the rural region age, educational level, marital status and income showed significant differences (all *P *<* *0.001). No difference concerning gender was found in the overall health literacy score nor within urban or rural regions.

**Table 2:** Urban–rural differences and associations in health literacy and associated factors

	HL total (*N *=* *1822)		HL in urban participants (*N *=* *1036)		HL in rural participants (*N *=* *786)	
	Mean (SD)	*P*-value	Mean (SD)	*P*-value	Mean (SD)	*P*-value
Region						
Urban	34.10 (8.07)	<0.001	–	–	–	–
Rural	35.73 (7.56)		–	–	–	–
Gender						
Male	34.85 (7.93)	0.887	33.92 (7.90)	0.987	36.21 (7.45)	0.393
Female	34.73 (8.03)		34.28 (8.22)		35.28 (7.64)	
Age group						
35–44	37.20 (7.28)	<0.001	36.01 (7.14)	<0.001	38.48 (7.04)	<0.001
45–54	36.62 (7.43)		35.42 (7.92)		38.19 (6.74)	
55–64	35.44 (7.82)		35.13 (8.21)		36.37 (6.68)	
65–74	32.20 (7.49)		31.81 (7.77)		32.71 (7.28)	
75+	29.74 (7.45)		29.35 (7.56)		30.38 (6.98)	
Educational level						
Basic	32.49 (7.66)	<0.001	31.38 (7.96)	<0.001	33.56 (7.18)	<0.001
Vocational	34.36 (7.99)		33.54 (8.02)		35.63 (7.61)	
Degree	37.85 (7.06)		37.78 (7.06)		37.93 (7.18)	
Marital status						
Married	35.35 (7.58)	<0.001	34.79 (7.94)	0.068	36.17 (7.01)	<0.001
Relationship	36.61 (7.23)		35.25 (7.40)		37.75 (6.57)	
Divorced	33.71 (7.69)		33.54 (7.32)		34.54 (7.32)	
Widowed	28.49 (8.07)		28.19 (8.42)		28.92 (7.04)	
Single	35.20 (8.68)		33.38 (8.64)		36.98 (9.01)	
Income						
Low	31.54 (7.96)	<0.001	30.77 (7.91)	<0.001	32.77 (7.74)	<0.001
Mid	35.97 (6.93)		35.29 (7.14)		36.99 (6.48)	
High	38.99 (6.96)		38.54 (7.22)		39.21 (6.84)	
Physical activity						
Low	34.25 (8.36)	<0.001	33.43 (8.71)	<0.001	35.21 (7.61)	0.246
Moderate	34.18 (7.38)		34.02 (7.40)		34.67 (7.68)	
High	36.72 (7.23)		35.56 (7.38)		38.23 (6.77)	

Health literacy (HL) levels (HLS-EU-Q16, range 0–50) and their distribution across urban and rural German participants (aged 35 years and older, *N *=* *1822) are presented. Data are shown with means (SD) and *P*-values.

The four steps of information processing and the three domains of health literacy were further analysed for urban–rural differences and are presented in table 3. In terms of the four steps of information processing, all values varied slightly at the 3.0 margin with accessing and appraising health information scoring the lowest. Participants in the rural region scored higher in each subcategory and also in the three domains (i.e. healthcare, disease prevention, health promotion). All differences were significant except for the subcategory appraising health information.

**Table 3:** Urban–rural differences in the four steps of information processing and the three domains of health literacy

	Urban participants (*N *=* *1036)	Rural participants (*N *=* *786)	
	Mean (SD)	Mean (SD)	*P*-value
Four steps of information processing			
Accessing health information	2.88 (0.60)	2.98 (0.62)	0.001
Understanding health information	3.18 (0.53)	3.31 (0.51)	<0.001
Appraising health information	2.90 (0.62)	2.94 (0.62)	0.236
Applying health information	3.12 (0.55)	3.22 (0.52)	<0.001
Three domains of health literacy			
Healthcare	3.06 (0.49)	3.17 (0.47)	<0.001
Disease prevention	3.01 (0.53)	3.11 (0.50)	<0.001
Health promotion	3.04 (0.55)	3.13 (0.56)	<0.001

Health literacy subscales and the three domains of health literacy across urban and rural German participants (aged 35 years and older, *N *=* *1822) are presented. Data are shown with means (SD) and *P*-values.

### Multivariate regressions

Two multiple regression models were specified to regress health literacy levels on its associated factors: all results are depicted in table 4. The first model demonstrates that living in an urban region, having vocational or basic education, having mid or low income, being widowed, or being moderate or low physically active were significantly negatively associated with health literacy. Model fit (*R*^2^) of this model was 0.197, therefore 19.7% of the variance can be explained. Thus, hypothesis 1 (participants in rural regions have lower scores in health literacy) must be rejected.

**Table 4:** Multiple multivariate regressions predicting health literacy in 1822 urban and rural German participants

*N *=* *1822	Model 1: Region, gender, age, educational level, income, marital status, physical activity				Model 2: Region, gender, age, educational level, Income, marital status, physical activity plus interaction terms			
	*B*	SE	*ß*	95% CI	*B*	SE	*ß*	95% CI
Region (Ref.: Rural)								
Urban	−1.72	0.34	−0.10	−2.39 to −1.05	−0.65	1.18	−0.04	−2.96 to 1.67
Gender (Ref.: Male)								
Gender (ref.: Male)	−0.34	0.34	−0.02	−1.00 to 0.32	−0.80	0.52	−0.05	−1.81 to 0.22
Age (continuous)								
Age (years)	−0.10	0.02	−0.17	−0.13 to −0.07	−0.14	0.02	−0.24	−0.18 to −0.09
Educational level (Ref: High)								
Vocational	−1.54	0.45	−0.10	−2.41 to −0.67	−0.58	0.68	−0.04	−1.92 to 0.76
Basic	−2.30	0.59	−0.10	−3.46 to −1.14	−1.18	0.87	−0.06	−2.89 to 0.53
Income (Ref.: High)								
Mid	−1.39	0.47	−0.12	−2.68 to −1.00	−1.53	0.72	−0.09	−2.94 to −0.11
Low	−4.44	0.54	−0.28	−5.50 to −3.38	−3.47	0.81	−0.22	−5.06 to −1.89
Marital status (Ref: Married)								
Relationship	0.10	0.50	0.00	−0.89 to 1.09	0.70	0.73	0.03	−0.73 to 2.14
Divorced	−0.04	0.64	−0.00	−1.30 to 1.21	−0.53	0.99	−0.02	−2.48 to 1.42
Widowed	−2.64	0.68	−0.10	−3.97 to 1.31	−2.76	1.03	−0.09	−4.77 to −0.75
Single	0.16	0.68	−0.00	−1.18 to 1.51	0.22	0.96	0.00	−1.66 to 2.10
Physical activity (Ref.: High)								
Moderate	−1.22	0.49	−0.07	−2.18 to −0.26	−2.42	0.80	−0.14	−3.99 to −0.86
Low	−1.14	0.44	−0.07	−2.00 to −0.30	−1.71	0.67	−0.11	−3.03 to −0.40
Interaction terms (Ref.: Rural)								
Gender × Region	–	–	–	–	0.78	0.68	0.05	−0.56 to 2.12
Age × Region	–	–	–	–	0.07	0.03	0.09	0.00 to 0.13
Educational level (Re.: High)								
Vocational × Region	–	–	–	–	−1.63	0.90	−0.10	−3.40 to 0.14
Basic × Region	–	–	–	–	−1.89	1.18	−0.07	−4.21 to 0.43
Income (ref.: High)								
Mid × Region	–	–	–	–	−0.76	0.96	−0.04	−2.63 to 1.11
Low × Region	–	–	–	–	−1.73	1.09	−0.09	−3.87 to 0.41
Marital status (Ref.: Married)								
Relationship × Region	–	–	–	–	−1.12	1.02	−0.04	−3.11 to 0.87
Divorced × Region	–	–	–	–	0.79	1.30	0.02	−1.76 to 3.34
Widowed × Region	–	–	–	–	0.27	1.37	0.01	−2.41 to 2.95
Single × Region	–	–	–	–	−0.15	1.37	−0.00	−2.84 to 2.54
Physical activity (Ref.: High)								
Moderate × Region	–	–	–	–	1.84	1.01	0.09	−0.14 to 3.82
Low × Region	–	–	–	–	0.93	0.88	0.05	−0.80 to 2.67
Model fit: *R*^2^	**0.197**				**0.206**			

Multiple multivariate regressions on predicting health literacy of German participants (*N  *= * *1822) aged 35 years and older. Regressions are based on 95% CI and *P  *< * *0.05.

The second model showed that the associated factors of the single variables had the same tendencies as in model 1. Further, the interaction term age × region showed a significant association. Thus, older residents of urban regions showed a prediction of higher health literacy levels than their rural counterparts. *R*^2^ was 0.202 (20.2% of explained variance). Thus, there is a significant interaction effect and hypothesis 2 (there are interaction effects) must not be rejected in terms of interaction with region and age.

## Discussion

The aim of this study was (i) to analyse urban–rural differences in health literacy in a metropolitan area in Europe and (ii) to explore how associated factors interact with health literacy. As main findings, we could detect that health literacy was higher in rural region and that all associated factors except gender contributed significantly to prediction of health literacy. By looking closer on interaction terms, we found the interaction age × region to be significant.

### Urban–rural differences in health literacy

Present results indicate higher levels of health literacy in rural regions, a finding that is not in line with previous empirical work as Aljassim and Ostini demonstrated in a recent systematic review.^22^ The authors found higher levels of health literacy in urban regions in all but one study. None of the studies included in the review were conducted in Europe, but mostly in developing countries or USA. One explanation of our contrary finding could be that in those countries the urban–rural gap in terms of infrastructure, education, access to healthcare or wealth is a greater magnitude, which may lead to a great inequity in terms of health outcomes.^31^^,^^32^ In the present study, the rural region is located in the surroundings of Berlin, which is not only the capital but also the most populous city in Germany with 3.7 million residents within the city and about 6 million residents within the metropolitan area. Here, we can find a trend that wealthy and educated people move to the outskirts of Berlin to find a quieter place to live. Thus, differences in health literacy might be more dependent on sociodemographic factors than region in our study.

### Sociodemographic factors and health literacy

As demonstrated in other studies^24^^,^^33^ and a systematic review^22^ differences in health literacy levels could not be explained by region alone. In our study, we demonstrated negative associations of health literacy with urban region, older age, lower educational level, lower income, being widowed and low levels of physical activity. There was no association found with gender. Overall, the present results are in line with previous studies.^10^^,^^22^^,^^24^ Another noticeable relation was detected by focusing on marital status. Only being widowed had a negative association, whereas being single, divorced or married/in a relationship made no difference. The association between being widowed and low levels of health literacy was also found in other studies^15^^,^^34^; however, this interpretation must be considered carefully, as there were only 147 widowed responders in comparison to 1234 being married. Moreover, an undetected ageing effect could bias the results.

Further, we found an interesting interaction effect. Older residents of urban regions showed a higher prediction of health literacy levels than their rural counterparts. A possible explanation for this finding can be, that especially in urban region one can find a vast number of doctors and health facilities making it hard to settle in. Hence, older residents might be more accommodated than younger ones. Also, older rural residents can face barriers to access to healthcare as infrastructure and especially specialist care is unsatisfactory.^35^ An inequity that is well-known but could be worked on in the future, for instance by implementing community nurses or telemedicine in rural regions.^36^

### Strengths and limitations

Strengths of our study include large sample size and balanced proportion between urban and rural participants, which increased statistical power to detect urban–rural differences. We demonstrate good analytic use of secondary data and propose a model that can easily be replicable with other databases for public health purposes.

However, some limitations must be outlined too. First, the present analysis is based on pre-pandemic data which were collected with the primary intent of examining antibiotic knowledge among residents of Berlin and Brandenburg. Therefore, some health literacy-related aspects were missing, such as history of hospitalization or being diagnosed with a chronic disease. Further, information for younger people (aged 34 years and younger) is missing, as they were not included in the study sample and the period of data collection was pre-pandemic. However, we do not believe, that our found associations are affected by the COVID-19 pandemic to a large amount, as a study in Germany found only slight changes in health literacy during the COVID-19 pandemic.^10^ Nonetheless, future research should focus on the lasting effects of the COVID-19 pandemic on health literacy. Also, the underlining data are cross-sectional, and hence there are no causalities that we can interpret.

In addition, our classification of urban and rural regions is based on participants’ ZIP codes and contextualized in LAU levels for European comparison. However, this classification only includes geographic aspects based on large databases and does not allow individual allocation. For instance, residents living in the outmost part of a large city with poor infrastructure would still be categorized as urban and residents in the centre of a smaller city with many amenities as rural. A further limitation is the dichotomous classification of urban and rural, which—although commonly performed—undermines areas or regions that are not as easily allocated.

However, these limitations are not only a weakness of our study but a known challenge in urban–rural research as the definitions and measurements of urban residence are still inherent and thus very hard to compare.^37^

### Implications for research and practitioners

More research should be done on further investigating the relationship of urban and rural regions and health literacy, especially in high-income countries, where rurality might even be an asset for health literacy. As rurality or urbanity alone does not appear as the substantial factor, more focus could be placed on the environmental factors at an individual level, such as infrastructure, access to healthcare or green ratio. Incorporating geographic information system, technologies in urban–rural health research could be one way to further investigate this matter. Also, urban–rural health research should agree on one distinct definition for urbanity and rurality (and regions that are in between) to make comparison and transfer easier. One step in the right direction is the Global Human Settlement Layer, a worldwide classification—built on the DEGURBA-classification—to compare cities by incorporating various databases.^38^ It is cogitable that rural areas will be incorporated too.

Further, the negative association of health literacy scores and being widowed stands out in various studies as a secondary finding with mainly limited validity due to small sample sizes, and hence, more focus can be put on this special vulnerable group. The findings of our study also indicate that older rural residents are at disadvantage in terms of health literacy, and limited access to healthcare could be one explanation but has to be further examined.

Practitioners and policymakers have known for a long time that health literacy plays a major role in a healthy society as seen in approaches such as the national action plan health literacy in Germany.^39^ The findings of our study underline the complexity of health literacy and that sociodemographic and environmental factors as well as health behaviour-related aspects should be taken into account in policy approaches. Also, researchers should critically follow the evolution of chatbots based on large language models and their impact on the overall health sector and health literacy.

## Conclusion

Health literacy is considered one key to a healthy society. The findings of this study show once more the multi-layered nature of health literacy and that the interplay of associated factors is not been fully explored yet. Although levels of health literacy cannot be drawn on region alone, environmental factors play an important role as they interact with other associated factors. Therefore, the focus on health literacy should be emphasized not only in research but also in societies in general.

## Data Availability

All data used for these analyses can be found here: https://osf.io/shd8t/files/osfstorage/640cd180c74723089710b166 Key points There has only been few research on urban–rural differences in health literacy in high-income countries. In contrast to low-income countries, health literacy was found to be higher in rural region compared to urban region. Age, income and education have the highest association with health literacy. Being widowed is negatively associated with health literacy scores. Gender made no difference. When focusing on urban–rural differences in health literacy interactions with associated factors should always be considered. We found older residents in urban regions to have higher levels of health literacy.
